# Cannabinoid Therapeutics in Chronic Neuropathic Pain: From Animal Research to Human Treatment

**DOI:** 10.3389/fphys.2021.785176

**Published:** 2021-11-30

**Authors:** Raquel Maria P. Campos, Andrey F. L. Aguiar, Yolanda Paes-Colli, Priscila Martins Pinheiro Trindade, Bruna K. Ferreira, Ricardo A. de Melo Reis, Luzia S. Sampaio

**Affiliations:** Laboratório de Neuroquímica, Instituto de Biofísica Carlos Chagas Filho (IBCCF), Centro de Ciências da Saúde, Universidade Federal do Rio de Janeiro, Rio de Janeiro, Brazil

**Keywords:** neuropathic pain, endocannabinoid, cannabidiol, THC, cannabis

## Abstract

Despite the importance of pain as a warning physiological system, chronic neuropathic pain is frequently caused by damage in the nervous system, followed by persistence over a long period, even in the absence of dangerous stimuli or after healing of injuries. Chronic neuropathic pain affects hundreds of millions of adults worldwide, creating a direct impact on quality of life. This pathology has been extensively characterized concerning its cellular and molecular mechanisms, and the endocannabinoid system (eCS) is widely recognized as pivotal in the development of chronic neuropathic pain. Scientific evidence has supported that phyto-, synthetic and endocannabinoids are efficient for pain management, while strong data arise from the therapeutic use of Cannabis-derived products. The use of medicinal Cannabis products is directed toward not only relieving symptoms of chronic pain, but also improving several aspects of patients’ welfare. Here, we review the involvement of eCS, along with other cellular and molecular elements, in chronic neuropathic pain pathology and how this system can be targeted for pain management.

## Chronic Neuropathic Pain

Chronic pain is classified by the International Association for the Study of Pain (IASP) as a pain that lasts more than 3 months, even after its primary cause is cured (Raja et al., 2020). One of the main types of chronic ache is neuropathic pain, that occurs when pain is caused by a lesion or disease of the somatosensory nervous system (Raja et al., 2020). Chronic neuropathic pain has several causes, such as the use of medicines (chemotherapy drugs, for example), metabolic diseases (such as diabetic neuropathy), demyelinating diseases (for instance, multiple sclerosis) and mechanical injuries (Meacham et al., 2017; Alles and Smith, 2018). The epidemiology of this disease varies across the globe, but it is estimated that 7–10% of all adults worldwide suffer from chronic neuropathic pain (van Hecke et al., 2014; Mücke et al., 2018). The main symptoms consist of spontaneous burning pain, numbness, and hyperalgesia (increased pain perception of noxious stimuli) and allodynia (pain hypersensitivity to normally innocuous stimuli) (Rani Sagar et al., 2012; Meacham et al., 2017; Alles and Smith, 2018). Patients may also experience social and economic consequences, since it is highly uncomfortable to conduct routine tasks while feeling pain. The physical and social impairment, along with the daily pain, can occasionally lead to depression (Knaster et al., 2012; Radat et al., 2013; Pitcher et al., 2019).

**FIGURE 1:**
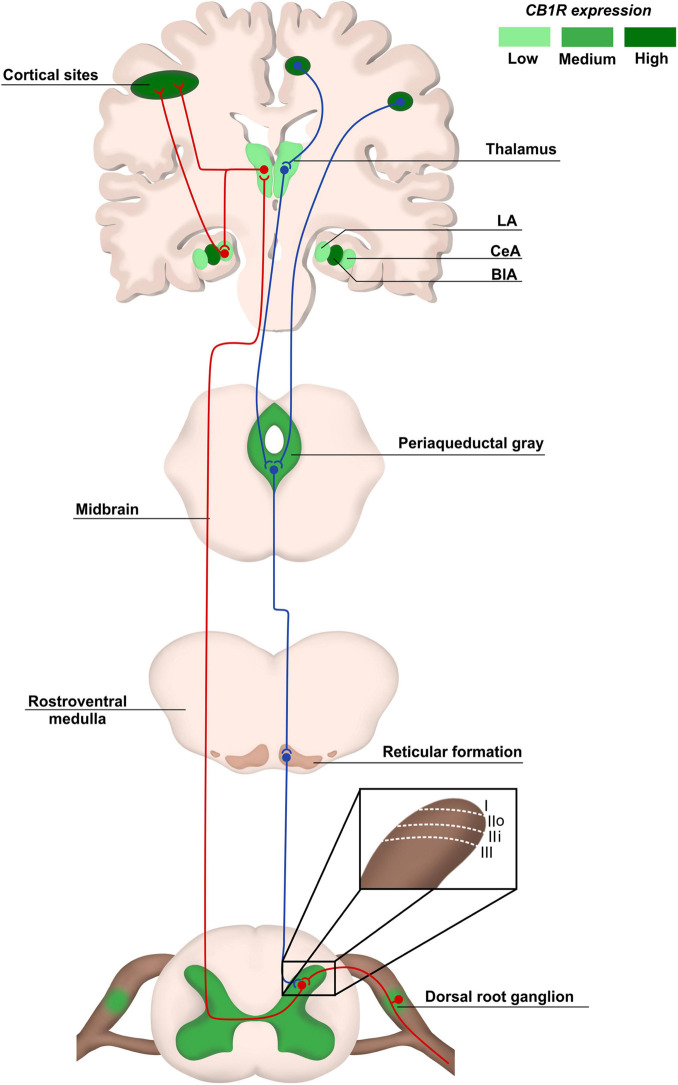
Pain anatomical pathways and CB1R expression. Ascending pain pathways (in red) are carried from the body periphery through sensory neurons of dorsal root ganglia (DRG) that synapse mainly with laminaes I to III in the dorsal spinal cord. Projection neurons make connections with brain areas such as thalamus and cortex. The descending pathways (in blue), responsible for pain modulation, involve areas such as the periaqueductal gray matter and amygdala, ending in the dorsal spinal cord. The CB1R distribution is heterogeneous in pain pathway areas, being more concentrated in regions such as cortex and Central Amygdala (CeA). LA, lateral amygdala; CeA, central amygdala; BlA, basolateral amigdala.

Physiological pain pathways include the peripheral and central nervous system (PNS and CNS, respectively), and the pain matrix revealed by neuroimaging in the last two decades is formed by central areas responsible for the process of pain (Legrain et al., 2011; Figure 1). Here, we focus on the plasticity in the spinal cord, particularly in the dorsal horn, due to its key role as a central integrator of afferent sensory information, besides being a region where significant part of pain processing occurs (Rani Sagar et al., 2012; West et al., 2015; Alles and Smith, 2018).

## Anatomical, Cellular, and Molecular Elements of Pain Processing in the Spinal Cord

The spinal cord is protected by the vertebral column and it is involved in motor and sensory processing, in addition to integrating the body with the brain through different pathways. Anatomically, the spinal cord is divided into an external white matter and an internal gray matter. The latter is subdivided in 10 laminae going from dorsal to ventral spinal cord, which differ from each other based on inputs received and neuron types (Rexed, 1952).

The dorsal horn consists of laminae I to VI, and receives information mostly from sensory neurons located in the dorsal root ganglia (DRG). The DRG neurons transduce mechanical, thermal or nociceptive information and can be classified as Aδ, C, or Aβ fibers (Rexed, 1952; Le Pichon and Chesler, 2014; Alles and Smith, 2018). Lamina I receives noxious, mechanical and thermal inputs from Aδ and C fibers. Lamina II consists of two zones: the outer zone, which receives inputs from C fibers, and the inner zone, receiving information from Aδ and C fibers. Aδ and Aβ fibers connect with other neurons in Laminae III to V carrying tactile and pressure information. Lamina VI receives sensory information from muscle spindles, consisting mostly of propriospinal neurons (Figure 1). All laminae have a high number of inhibitory GABAergic and glycinergic interneurons that help modulate sensory inputs. Also, it is important to highlight that most of the laminae in the dorsal horn make connections with neurons from different brain regions through ascendant and descendant pathways (West et al., 2015; Alles and Smith, 2018; Figure 1).

The synapses between Aδ, Aβ, and C fibers and spinal cord neurons are excitatory, having glutamate as neurotransmitter (West et al., 2015; Alles and Smith, 2018). Glutamate release from sensory fibres is regulated by inhibitory interneurons present in all laminae of the dorsal horn through γ-aminobutyric acid (GABA) or glycine release, modulating noxious transmission (West et al., 2015). The sensory information travels through different pathways, such as the spinothalamic tract, to different brain areas known as pain matrix, which includes the thalamus, the anterior cingulate cortex, the periaqueductal gray matter, the amygdala and others (D’Mello and Dickenson, 2008; Cohen and Mao, 2014; Colloca et al., 2017; Figure 1). Pain modulation is a top-down process: after information processing in higher brain centers, neurons that form the descendant pathways make synapses in the dorsal horn, releasing serotonin, GABA and glycine (D’Mello and Dickenson, 2008; Ossipov et al., 2010; West et al., 2015; Colloca et al., 2017; Figure 1).

## Endocannabinoid System in Physiological Pain Processing

**FIGURE 2:**
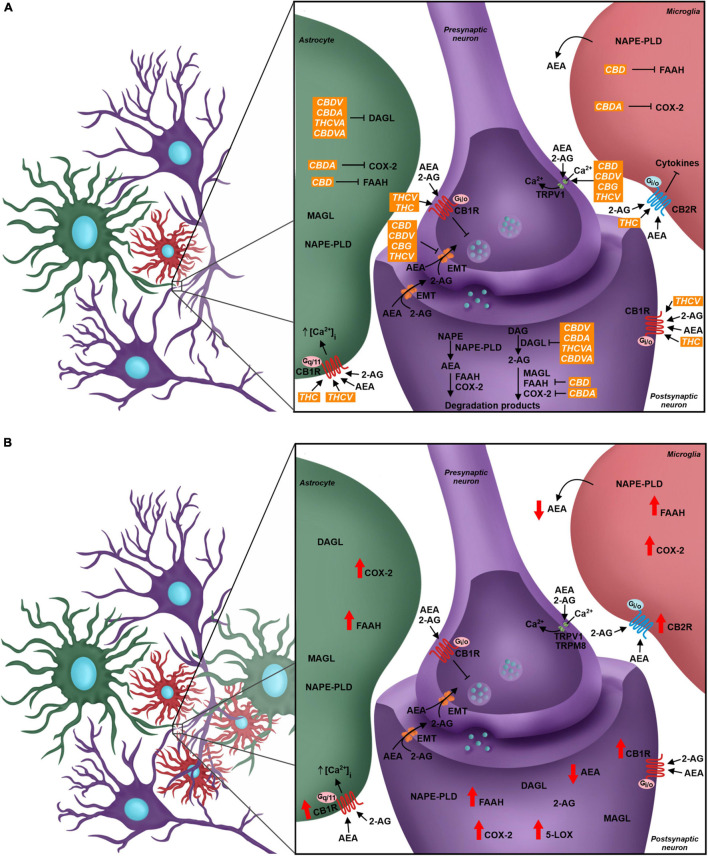
The role of the endocannabinoid system (eCS) in the quadripartite synapse, its modulation by phytocannabinoids and alterations due to Neuropathic Pain. **(A)** Neurons, astrocytes, and microglial cells have the eCS components, and the endocannabinoid signaling through CB1R and CB2R leads to different outcomes in each cell. The presynaptic neuron expresses CB1R, TRPV1, TRPM8, and the endocannabinoid membrane transporter (EMT). Receptors are targeted by endocannabinoids (AEA and 2-AG). CB1R modulation activates signaling cascades that inhibit Ca^2+^ intracellular influx, which decreases the fusion of intracellular vesicles with the neuron membrane, changing the neurotransmitter release flow. The postsynaptic neuron also presents, besides the receptors, all the elements of the ECS, such as the AEA and 2-AG synthesis enzymes, respectively NAPE-PLD and DAGL, and the degradation enzymes, FAAH, MAGL, and other enzymes such as COX2. In green: Astrocyte takes part in the synapse and expresses different elements of the eCS such as endocannabinoids’ synthesis and degradation enzymes and cannabinoid receptors, where the activation of CB1R may favor the influx of Ca^2+^ ions. Microglia expresses components of the eCS; the CB2R expression is higher than CB1R, and its modulation is linked to the production and secretion of different cytokines. Phytocannabinoids modulate the eCS through many targets. THC and THCV are CB1R agonists, while CBD, CBDV, CBG, and THCV are TRPV1 agonists. The EMT transporter is the pharmacological target of the phytocannabinoids CBD, CBDV, CBG, and THCV. The phytocannabinoids also act over enzyme activity - CBD inhibits FAAH, CBDA inhibits COX-2 and CBDV, CBDA, THCVA and CBDVA inhibit DAGL. **(B)** In the Neuropathic Pain scenario, there is glial reactivity, leading to the increase of astrocyte and microglia next to neurons, especially in the dorsal spinal cord. The eCS is modulated and the levels of expression of your components change. There is a higher expression of CB1R and CB2R in neurons and glial cells. Enzymes such as FAAH, COX-2, and 5-LOX also increase their expression, and in result, there is a decrease of AEA levels and increase of pro-inflammatory mediators. CBD, cannabidiol; THC, tetrahydrocannabinol; CBDV; cannabidivarin; CBDA, cannabidiolic acid; THCVA, tetrahydrocannabivarinic acid; CBDVA, cannabidivarinic acid; THCV, tetrahydrocannabivarin; CBG, cannabigerol.

The endocannabinoid system (eCS) main components are the G protein-coupled cannabinoid receptors CB1 (CB1R) and CB2 (CB2R), the endocannabinoids (eCBs) for example anandamide (AEA) and 2-arachidonoylglycerol (2-AG), and the enzymes involved in their metabolism, such as fatty acid amino hydrolase (FAAH) and monoacylglycerol lipase (MAGL), responsible for the degradation of AEA and 2-AG, respectively (Howlett et al., 2002; Figure 2). The eCS is an on-demand system and heterogeneously present in different structures of the CNS and PNS, including important regions of pain processing, such as the DRGs, spinal cord, thalamus, amygdala and others (Tsou et al., 1998; Farquhar-Smith et al., 2000; Katona et al., 2001; Starowicz and Finn, 2017; Finn et al., 2021; Figure 1).

In relation to pain modulation in the dorsal spinal cord, the eCS acts as a regulator of the synaptic transmission in the DRGs. CB1R is expressed in the presynaptic sensory fibers of trigeminal ganglion and dorsal root ganglion, besides the nerve endings of primary sensory neurons in dermis, whose afferent fibers conduct nociception (Salio et al., 2002; Price et al., 2003; Veress et al., 2013; Zou and Kumar, 2018). Following the release of neurotransmitters, glutamatergic receptors are activated in the postsynaptic terminal, inducing Ca^2+^ influx and its increased concentration inside the cell. Therefore, higher levels of intracellular Ca^2+^ promotes activation of enzymes responsible for eCBs synthesis, mostly AEA and 2-AG, which are then released into the synaptic cleft and bind to CBRs in the presynaptic terminal. CBR activity induces blockade of voltage-gated Ca^2+^ channels presynaptically and inhibits adenylate cyclase, decreasing levels of cAMP and triggering the signaling cascade involved in synaptic plasticity, besides modulating sensory transmission through this feedback mechanism in the dorsal horn (Shen et al., 1996; Mecha et al., 2015; Figure 2A). This was demonstrated by the development of thermal hyperalgesia and blockage of inhibition of evoked excitatory postsynaptic currents (eEPSC) in laminae II neurons in mice after administration of CB1R antagonists (Richardson et al., 1997; Yang et al., 2016).

Another important component associated with the eCS is the transient receptor potential family (TRP). The TRP cation channel subfamily V member 1 (TRPV1) has a relevant role in nociceptive transduction in the PNS since it is activated, opening a Ca^2+^ channel and increasing this ion concentration inside the neurons, by heat, acidic substances and capsaicin, besides being expressed in the soma and nerve terminals of sensory neurons from DRG (Caterina et al., 1999; Immke and Gavva, 2006; Lauria et al., 2006). The TRP cation channel subfamily M member 8 (TRPM8) and subfamily A member 1 (TRPA1) also act as nociceptors and they are activated by menthol/cold temperatures and noxious cold/pungent compounds, respectively (Storozhuk and Zholos, 2018). Although TRPV1 and TRPM8 were not initially considered eCS-related receptors, they are directly influenced by different endocannabinoids, such as AEA, since this molecule is an agonist for the former and antagonist for the latter (Zygmunt et al., 1999; De Petrocellis et al., 2007; Figure 2A).

Clinical evidence for the role of the eCS in pain management was reported based on a serendipitous case of a Scottish patient (Habib et al., 2019). Authors described the clinical data from a woman submitted to orthopedic surgery, a procedure recognized for being associated with severe pain, with no need for analgesics. The same patient also had a long clinical history of cuts and burns without any sensation of pain. Genetic investigation revealed a deletion in the gene responsible for FAAH transcription, which led to reduced degradation and higher levels of AEA in peripheral blood and probably other organs.

The major eCS components are also present in glial cells, such as astrocytes and microglia, even though CB2R is more expressed than CB1R in microglia, as also shown in other immunological cells, such as lymphocytes and neutrophils (Hegyi et al., 2009; Greineisen and Turner, 2010; Maccarrone et al., 2015). In astrocytes, CB1R-mediated signaling promotes increase of intracellular Ca^2+^ levels, while activation of microglial CB2R maintains the resting state or anti-inflammatory polarity of this cell type (Greineisen and Turner, 2010; Mecha et al., 2015; Figure 2A).

## Cellular and Molecular Changes in the Spinal Cord Associated With Chronic Neuropathic Pain

The nervous system is plastic, with the ability to change and readapt in response to environmental stimuli. In this context, maladaptive plasticity processes can result in malfunction of the nervous system physiology (Kuner and Flor, 2017; Meacham et al., 2017). For instance, hyperalgesia is related to neuronal hyperexcitability triggered by cytokines and inflammatory mediators released in the periphery or in the spinal cord (Colloca et al., 2017; Kuner and Flor, 2017; Meacham et al., 2017). This is followed by a decrease of GABA- and glycine-mediated neurotransmission, caused not only by the reduction of their release, but also because of inhibitory interneurons apoptosis (Moore et al., 2002; Janssen et al., 2011; Foster et al., 2015). The decrease of GABA-mediated signaling reduces presynaptic inhibition, especially in Lamina II, which allows Aβ fibers to communicate with neurons in Lamina I, therefore contributing for allodynia (Alles and Smith, 2018).

The central sensitization of the spinal cord, which is the increased responsiveness of nociceptive neurons in the CNS when compared to normal threshold, may occur for several reasons, one of them being the dysfunction of glutamate signaling (Meacham et al., 2017). The expression of glutamate transporters is downregulated after PNS injury, increasing the availability of glutamate to their receptors and decreasing neuron-firing threshold (Sung et al., 2003). Changes in the expression of voltage-gated calcium channels, such as the upregulation of α2δ-1 subunit, also contribute to neuron hyperexcitability by increasing Ca^2+^ permeability to the intracellular medium (Li et al., 2004; D’Arco et al., 2015).

The unbalanced synaptic communication in the dorsal horn of the spinal cord is one of the main causes for chronic neuropathic pain consolidation. However, synapses not only contain pre- and postsynaptic elements, but also the participation of glial cells, such as astrocytes and microglia, which have their physiological state shifted and contribute to this pathology, similarly to neurons. After PNS injury, it is well-known that astrocytes and microglial cells in the spinal cord show increased reactivity, identifiable by changes in their morphology and secreted molecules (Burgos et al., 2012; Alles and Smith, 2018). After an aversive stimulus, these cells produce pro-inflammatory mediators, such as interleukins -17, -1β, -6 (IL-17, IL-1β, and IL-6), and Tumor Necrosis Factor-α (TNF-α), which establish an inflammatory environment involved in the maintenance of chronic neuropathic pain (Wieseler-Frank et al., 2005; Stemkowski et al., 2017). Part of these molecules are chemoattractant for immune cells, which explains the infiltration of T cells (Choi et al., 2015; Sun et al., 2017). Indeed, several studies describe the correlation between lymphocyte invasion in the spinal cord and the development of chronic pain, also by the production and release of cytokines as IL-17 by this cell type (Kleinschnitz et al., 2006; Davoli-Ferreira et al., 2020).

Glial cells are also involved in hyperalgesia and allodynia generation. Astrocytes potentiate glutamatergic signaling by reducing the expression of excitatory amino acid transporter 2 (EAAT2) in these cells, which promotes increased glutamate concentration externally to the neuron (Cata et al., 2006). In addition, the contents of purinergic receptors are increased in the microglial cytoplasmic membrane. The continuous adenosine triphosphate (ATP) release from injured and stressed cells in the microenvironment induces constant stimulation of microglia reactivity, proliferation and pro-inflammatory polarity (Tsuda et al., 2013; Peng et al., 2016; Alles and Smith, 2018). In fact, P2X purinoceptor 4 (P2X4) stimulation in the spinal cord of non-injured adult rats is sufficient to induce allodynia (Tsuda et al., 2003; Niu et al., 2017).

## Endocannabinoid System as Target for Chronic Neuropathic Pain Treatment

As previously mentioned, the unbalance of eCS physiological signaling can induce chronic neuropathic pain symptoms (Richardson et al., 1997; Yang et al., 2016). The eCS components are highly susceptible to molecular alterations, and such events are observed in chronic neuropathic pain pathogenesis, both in the CNS and PNS (Rani Sagar et al., 2012; Starowicz and Finn, 2017). Indeed, it has been described that the expression of CB1R and CB2R, the eCB synthesis machinery and the expression of FAAH are increased in the spinal cord of animals submitted to murine models of chronic neuropathic pain (Zhang et al., 2003, 2010; Guindon et al., 2013; Davis, 2014; Malek et al., 2014). Although there is elevation in eCBs synthesis, activation of FAAH and alternative catabolic pathways, involving cytochrome p450 (CYP), cyclooxygenase-2 (COX-2) and lipoxygenases, increase AEA degradation and generates inflammatory mediators, such as prostaglandins, which influence neuron excitability (Kozak et al., 2002; Snider et al., 2009; Chouinard et al., 2011; Rani Sagar et al., 2012; Mecca et al., 2021). The reduction of AEA and CB1R-mediated negative feedback in excitatory synapses contribute to the hyperalgesia mechanism (Figure 2B).

The fact that the eCS is involved in the pathophysiological state of pain makes this system a valid target for chronic neuropathic pain treatment. Nowadays, treatments for chronic neuropathic pain consist of four lines of therapies that are chosen according to the patient’s condition (Table 1), with opioids being the most used (Attal et al., 2010; Rani Sagar et al., 2012; Moulin et al., 2014; Finnerup et al., 2015).

**TABLE 1:** The four lines of treatment for chronic neuropathic pain.

Treatment	Mechanism of action	Line of treatment	References
Gabapentinoids	α2δ2-1 subunit of voltage-gated Ca^2+^ channels ligant	First-line treatment	Attal et al., 2010; Moulin et al., 2014; Hennemann-Krause and Sredni, 2016; Colloca et al., 2017
Tricyclic antidepressants	Inhibitors of Noradrenaline/Serotonin uptake systems	First-line treatment	Attal et al., 2010; Moulin et al., 2014; Hennemann-Krause and Sredni, 2016; Colloca et al., 2017
Noradrenaline/serotonin reuptake inhibitors	Inhibitors of noradrenaline/serotonin reuptake systems	First-line treatment	Attal et al., 2010; Moulin et al., 2014; Hennemann-Krause and Sredni, 2016; Colloca et al., 2017
Weak opioids	Opioid receptors agonists	Second-line treatment	Attal et al., 2010; Moulin et al., 2014; Hennemann-Krause and Sredni, 2016; Colloca et al., 2017
Strong opioids	Opioid receptors agonists	Second-line treatment	Attal et al., 2010; Moulin et al., 2014; Hennemann-Krause and Sredni, 2016; Colloca et al., 2017
Cannabinoids	Endocannabinoid system modulators	Third-line treatment	Attal et al., 2010; Moulin et al., 2014; Colloca et al., 2017
Selective serotonin reuptake inhibitors (SSRI)	Inhibitors of selective serotonin reuptake system	Fourth-line treatment	Moulin et al., 2014
Botulinum toxin	Inhibitors of acetylcholine release	Fourth-line treatment	Moulin et al., 2014
Methadone	Opioid and NMDA receptors	Fourth-line treatment	Moulin et al., 2014
Lamotrigine	Inhibitors of voltage-gated Na^+^ and Ca^2+^ channels	Fourth-line treatment	Moulin et al., 2014
Lacosamide	Slow inactivation of voltage-gated Na^+^ channels	Fourth-line treatment	Moulin et al., 2014
Tapentadol	Opioid receptors agonist and inhibitor of noradrenaline uptake system	Fourth-line treatment	Moulin et al., 2014
Topical lidocaine	Sodium channel blocker	Fourth-line treatment	Moulin et al., 2014
Topical capsaicin	TRPV1 receptor desensitization	Fourth-line treatment	Moulin et al., 2014

Despite the variety of treatments, there are no effective pharmacotherapeutic strategies for mitigating chronic neuropathic pain, in addition to patients that do not respond properly to treatments and are resistant to current medicines available (Dworkin et al., 2010; Yekkirala et al., 2017). Moreover, there is a huge opioid crisis with severe consequences centered on drug addiction, respiratory depression and death by overdose (Moore and McQuay, 2005; Fornasari, 2017; CDC, 2021; WHO, 2021). The lack of a solid treatment with few or no side effects makes the pharmaceutical industry avid to pursue new alternatives for these patients, and the modulation of eCS has been considered a promising tool.

CB1R/CB2R agonists and antagonists have been described as a valuable option to successfully modulate the eCS in chronic neuropathic pain animal models, bringing an alternative of treatment for patients that do not respond well to other pharmacological therapies. In addition, an indicative that modulating cannabinoid receptors could be a good alternative instead of opioids is the increased density of cannabinoids receptors in the spinal cord, compared to opioid receptors (Hohmann et al., 1999), and high/moderate expression of cannabinoid receptors in brain areas responsible for pain modulation, such as cortex, amygdala and periaqueductal gray matter, at least in rodents (Befort, 2015; Figure 1).

In animal models of chronic neuropathic pain, the administration of the synthetic cannabinoid CP 55,940, a CB1R agonist, terminated thermal hyperalgesia and decreased mechanical allodynia, evaluated by hot plate test and von Frey test, respectively (De Vry et al., 2004; Scott et al., 2004; Romero-Sandoval and Eisenach, 2007). A single administration of WIN55, 212-2, a mixed CB1R/CB2R-receptor agonist, 7 days after nerve ligation (a murine model of chronic neuropathic pain), reduced cold allodynia and thermal hyperalgesia symptoms, evaluated by acetone and hot plate test, respectively (Bridges et al., 2001; Rahn et al., 2007). The use of WIN55, 212-2 also improved mechanical allodynia at von Frey test in chemotherapy-induced chronic neuropathic pain, when animals presented behavior similar to those treated with opioids (Rahn et al., 2007; Burgos et al., 2012). At the cellular level, this agonist reduced glial reactivity and expression of inflammatory mediators, such as IL-6 and TNF-α (Burgos et al., 2012). It is important to notice that the combination of WIN55, 212-2 with selective CB1R and CB2R antagonists, SR141716 and SR144528, respectively, reversed the allodynia improvement, evaluated by von Frey test, demonstrating that both cannabinoid receptors are directly involved in these mechanisms and can be targeted for treatment purposes (Rahn et al., 2007). In addition, injection of JWH133 or JWH015, CB2R agonists, decreases mechanical allodynia after partial nerve ligation (Romero-Sandoval and Eisenach, 2007; Romero-Sandoval et al., 2008; Yamamoto et al., 2008). As described, CB2R is mostly expressed by microglial cells, which have their migration, proliferation and polarity modulated by cannabinoid receptor activation (Hegyi et al., 2009; Stella, 2009; Greineisen and Turner, 2010). Alternatively, CB2R activation by AM1241 decreases the expression of purinergic receptors P2Y, which is upregulated in microglia of chronic neuropathic animals, and decreases nuclear factor κ B (NF-κB) and p38 mitogen-activated protein kinase (p38 MAPK) phosphorylation, both involved in microglial activation and inflammatory response (Niu et al., 2017). The addition of 2-AG and AEA in primary microglial cell cultures increased the expression of both cannabinoid receptors and arginase-1, a marker for M2 microglia polarity, which is associated with pro-healing and anti-inflammatory responses (Mecha et al., 2015). The direct administration of AEA also led to better sensorial behavior in neuropathic murine animals, increasing mechanical and thermal threshold, evaluated by von Frey and hot plate tests (Guindon and Beaulieu, 2006; Desroches et al., 2008).

Moreover, some studies referred to the catabolic enzyme FAAH as an alternative target to modulate eCB levels. Intraperitoneal administration of the FAAH blocker URB597 and MAGL blocker JZL184 led to an increase in the mechanical threshold and a reduction of cold allodynia, in von Frey and acetone tests, in chronic neuropathic rats, respectively (Clapper et al., 2010; Guindon et al., 2013). The oral administration of another FAAH blocker, ST4070, also produced the same improvement in several animal models of chronic neuropathic pain, such as those induced by chemotherapy drugs and diabetes (Caprioli et al., 2012). Changes in animal behavior due to pain is probably correlated to the increased availability of eCB and other bioactive lipids, for instance AEA and palmitoylethanolamide (PEA), respectively (Caprioli et al., 2012).

After the enlightenment of eCS participation in pain modulation in physiological and pathological states, researchers started to investigate if classical analgesics mechanisms could involve the eCS. Regarding the effects of dipyrone, a common non-steroidal anti-inflammatory drug widely used primarily as an analgesic and antipyretic, the exact action mechanisms remain controversial. Studies on mice suggest that dipyrone-induced suppression of thermal antinociceptive, hypothermic and locomotor activity is mediated by a CB1R/CB2R-independent mechanism (Schlosburg et al., 2012). On the other hand, AM251, a CB1 antagonist, reversed the effects of dipyrone on locomotor activity, cataleptic response and thermal analgesia (Crunfli et al., 2015). Both AM251 and capsazepine, a TRPV1 antagonist, favored the decrease in body temperature caused by dipyrone. However, the CB2 receptor antagonist AM630 did not alter the hypothermic response to dipyrone (Crunfli et al., 2015). These results suggest that the eCS role, especially CB1R-mediated, in the analgesic effect of dipyrone is still a matter of debate.

Although the evidence in animal models is promising and suggests that inhibitors of catabolic enzymes might be a way of treating chronic neuropathic pain, clinical trials did not show the same outcome. In January 2016, it came to public attention that a drug named BIA10-2474, a FAAH inhibitor, led to severe adverse events in some volunteers in the clinical trial, in which five people had to be hospitalized, two had brain damage and one died (Mallet et al., 2016). Nowadays, in clinical practice, the synthetic cannabinoids used to treat neuropathic chronic pain are Dronabinol (Marinol^®^ – Solvay Pharmaceuticals) and Nabilone (Cesamet^®^ – Meda Pharmaceuticals), both having a chemical formula based on Δ^9^-tetrahydrocannabinol (Δ^9^-THC) (Stasiulewicz et al., 2020). Although these compounds may improve symptoms related to chronic neuropathic pain, some side effects such as euphoria, dysphoria, sleep disturbance and disorientation may occur. Diversely, the use of medicinal cannabis shows better results and less adverse events (Cannabis-In-Cachexia-Study-Group et al., 2006; Frank et al., 2008; Narang et al., 2008; Aviram and Samuelly-Leichtag, 2017).

## Medicinal Cannabis in Animal Models and Clinical Evidence

*Cannabis* sp. has been used to treat several pathologies, including pain episodes, since ancient China (Bonini et al., 2018). The main components in *Cannabis* sp. are the phytocannabinoids, and more than 100 of these compounds have been described thus far. Even though the most investigated phytocannabinoids are cannabidiol (CBD) and Δ^9^-THC, the latter known for its psychoactive effect, several other phytocannabinoids such as cannabidivarin (CBDV), Δ^9^-tetrahydrocannabivarin (THCV) and their acidic forms are also being considered for therapeutic purposes (Di Marzo and Piscitelli, 2015; Cristino et al., 2020).

Phytocannabinoids act over several targets to modulate the eCS. For instance, while THC acts as CB1R and CB2R agonist, and THCV is a CB1R antagonist and TRPV1 agonist, CBD and CBDV inhibit eCB-degradation enzymes FAAH and MAGL (Di Marzo and Piscitelli, 2015; Cristino et al., 2020). Some phytocannabinoids also act in other neurochemical systems. For example, CBD can act over the serotoninergic and glycinergic receptors present in neurons, which are involved in pain processing, besides acting as TRPV1 and TRPM8 antagonist (Russo et al., 2005; De Petrocellis et al., 2008; Ahrens et al., 2009; García-Gutiérrez et al., 2020; Figure 2A).

Despite the recent statement from the IASP not encouraging the use of cannabinoids for pain treatment based on lack of quality researches reinforcing its safety and efficacy (IASP Presidential Task Force on Cannabis and Cannabinoid Analgesia, 2021), the positive outcomes and advantages over other pharmacological tools are compelling in animal research and clinical trials. For instance, Abraham et al. (2020) demonstrated in animals a phenomenon similar to what happens with patients. Neuropathic rats had a good response to morphine treatment, but the effect did not last up to 22 days after induction of pain, followed by drug-resistance. However, this effect was not observed in THC- and CBD-treated rats.

Tetrahydrocannabinol is known as the main analgesic compound from *Cannabis* sp. (Russo, 2008; Maayah et al., 2020). Several studies described a reduction of allodynic and hypersensitive behavior in animals, based on von Frey and hot plate tests results, after THC administration (Harris et al., 2016, 2019; Abraham et al., 2020). In addition to the fact that THC is a CB1R and CB2R agonist, reducing neurotransmitters release by neurons, especially glutamate, it also acts as TRPM8 antagonist and TRPA1 agonist (De Petrocellis et al., 2008; Storozhuk and Zholos, 2018). Another mechanism of action is inhibiting COX-2, which leads to increased levels of AEA and decreased levels of prostaglandins, whose therapeutic properties in chronic neuropathic pain pathology are the reduction of pro-inflammatory signaling, and probably, the decrease in glial and immunological cells response (Burstein et al., 1973; Ruhaak et al., 2011; Figure 2A).

CBD also contributes to decreased chronic neuropathic pain symptoms. Studies that investigated acute and chronic treatments of chronic neuropathic pain-induced rats with isolated CBD showed significantly increased mechanical and thermal threshold when compared to animals that received vehicle, evaluated by von Frey and hot plate tests (Xiong et al., 2012; Harris et al., 2016; King et al., 2017; Abraham et al., 2020; Silva-Cardoso et al., 2021). A plausible hypothesis that might explain how CBD improves the pathologic pain sensation is related to an increase in AEA levels due to FAAH inhibition and their agonist activity on TRPV1 agonist (Bisogno et al., 2001; Massi et al., 2008; Silva-Cardoso et al., 2021; Figure 2A). Silva-Cardoso et al. (2021) also demonstrated that CBD treatment decreased CB1R expression in pain matrix regions, which was up-regulated in animals submitted to chronic neuropathic pain models.

Furthermore, the combination of CBD and THC synergizes their positive effects and reduces THC side effects (Carlini et al., 1974; McPartland and Russo, 2001). In fact, the co-administration of CBD and THC decreases dysphoria, anxiety, panic attacks, and other psychoactive effects that THC may cause (Grinspoon and Bakalar, 1993; King et al., 2017). Authors also described that CBD competes with THC for the binding site of CYP2C19 enzyme in the liver and inhibits THC hydroxylation, which prolongs THC bioavailability, and therefore its effects (Jones and Pertwee, 1972; Benowitz et al., 1980).

The involvement of other compounds from *Cannabis* sp. in pain improvement have also been reported, for example, cannabinol (CBN) and cannabichromene (CBC), acting as CB2R agonist and inhibitor of cyclooxygenase, respectively (Burstein et al., 1973; Showalter et al., 1996; Figure 2A). Terpenes are another class of secondary metabolites present in *Cannabis* sp. with biological relevance and they may play a role in pain treatment, such as the terpenoid β-caryophyllene, which directly modulates the eCS as a CB2R agonist (Gertsch et al., 2008). In the chronic neuropathic pain animal model, the daily gavage of β-caryophyllene decreased nociceptive hyperalgesia and mechanical allodynia in a dose-dependent manner, as demonstrated by hot plate and von Frey tests. The treatment also reduced microglial reactivity and inflammatory response at spinal dorsal horn, probably due to β-caryophyllene-mediated increased expression of CB2R (Klauke et al., 2014; Segat et al., 2017). Other terpenoids participate in pain modulation through anti-inflammatory response. For instance, administration of β-myrcene increased thermal and nociceptive threshold either in healthy mice (Rao et al., 1990) or under inflammatory pain (Lorenzetti et al., 1991). Additionally, α-pinene also seems to control pain through anti-inflammatory pathways, decreasing cyclooxygenase-2 expression in an animal model of inflammatory pain (Li et al., 2016). Lastly, flavonoids, a group of chemical compounds present not only in *Cannabis* sp., but also in other plants, have anti-inflammatory properties and can decrease the release of pro-inflammatory cytokines from astrocytes and microglia (Gui et al., 2018; Nadipelly et al., 2018).

Several compounds present in the *Cannabis* sp. extract synergize with each other and might modulate their effects when compared to isolated phytocannabinoids (Carlini et al., 1974; Russo, 2008). The synergistic action of phytocannabinoids producing a potentiated pharmacological effect is called entourage effect (King et al., 2017). This property was described in several studies that reported higher efficacy of *Cannabis* sp. extract compared to administration of isolated CBD and THC in decreasing allodynia and hyperalgesia in rats, evaluated by hot plate and von Frey tests (Comelli et al., 2008; Casey et al., 2017; Harris et al., 2019). Indeed, Comelli et al. (2008) described that the improvement in hyperalgesia through the use of *Cannabis* sp. extract was not affected by the use of cannabinoid receptor antagonists, reinforcing the fact that phytocannabinoids and other compounds in the extract act synergistically modulating several receptors and pathways. Additionally, the administration of a 1:1 CBD:THC extract has been described to positively modulate CD4+ lymphocytes in the spleen and thymus of female rats submitted to a nerve cuffed model for chronic neuropathic pain, which suggests that *Cannabis* sp. extract may also modulate immune response in the CNS related to chronic neuropathic pain development (Linher-Melville et al., 2020).

Patients consume medical *Cannabis sp.* products through different administration routes, such as smoked, vaporized, oromucosal aerosol, oily extract and capsules, although some studies describe that inhaling procedures can present health risks for patients (Aviram and Samuelly-Leichtag, 2017; Maayah et al., 2020). As described in animal studies, tests showed that *Cannabis* sp. full extract, such as Sativex^®^, an oromucosal spray of 1:1 CBD:THC, was more effective for treating chronic neuropathic pain than synthetic THC, such as Dronabinol (Cannabis-In-Cachexia-Study-Group et al., 2006; Langford et al., 2013; Ferrè et al., 2016; Schimrigk et al., 2017). Nurmikko et al. (2007) coordinated a randomized, double-blind, placebo-controlled study to evaluate the analgesic properties of a THC:CBD (1:1) extract in 125 patients that had peripheral neuropathic pain symptoms. During the treatment with the extract, individuals continued with other analgesic medication previously prescribed. Sixty-three patients were in the placebo group, while 62 made use of the extract and were able to determine their own dose, although none of them had more than 48 spray doses per day. The group that was treated with the extract showed better results in pain scores, dynamic allodynia, punctual allodynia when compared to patients from the other group. An appendix of the study evaluated the same patients for 52 weeks and continued to observe the analgesic effect of the extract without dose adjustment or toxicity symptoms.

A more complete and recent study from the Germain Pain e-registry digital platform collected anonymous information related to the therapeutic approaches used for pain management (Ueberall et al., 2019). Using the answers from a Pain Detect Questionnaire 7 (PDQ7), patients were grouped with nociceptive, mixed and chronic neuropathic pain. Individuals used 1:1 CBD:THC Sativex^®^ oromucosal spray, 8–12 times/day, for 12 weeks. After 3 months of *Cannabis* sp. spray use, the pain intensity decreased at least half in 67.5% of the patients, having a better effect in neuropathic and mixed pain patients. Another important information from this study consists in improved patient’s welfare after *Cannabis sp.* treatment. This was measured by the Aggregated 9-Factor Symptom Relief (ASR-9) questionnaire. Fifteen percent of the individuals in the study improved at least 50% of all 9 factors and 56% of the patients improved at least 5 factors, such as stress, depression, well-being and anxiety, and again, the improvement was higher in neuropathic and mixed pain patients.

Another advantage to the use of Cannabis-derived products is the significant decrease or even elimination of other drugs, such as opioids, from the therapy scheme, known for having severe adverse effects. As described by Ueberall et al. (2019), a significant number of patients stopped using strong opioids as analgesic strategy 12 weeks after using *Cannabis sp.* extracts. Other clinical and animal researches describe similar results in opioid-based therapies after the use of *Cannabis* sp. extracts (Williams et al., 2008; Okusanya et al., 2020; Takakuwa and Sulak, 2020).

Together with the relief of symptoms, adverse effects are often described by patients under use of medicinal cannabis. The most common are gastrointestinal disorders, metabolism and nutrition disorders, increased appetite, sedation, fatigue, dry mouth, dizziness, and nausea (Nurmikko et al., 2007; Lynch et al., 2014; Ueberall et al., 2019). Few patients described adverse effects related to the psychoactive properties of THC. One patient described anxiety, panic attack, and confusion in the Lynch study (Lynch et al., 2014) and only 3.6% of patients described anxiety, confusion, and disorientation in the Germain Pain e-registry based study (Ueberall et al., 2019), while, in Nurmikko et al. (2007), no psychoactive symptoms were listed by the participants. In general, most of the adverse effects are mild to moderate and are directly related to the dosage of THC in the product used in these researchers, besides the modulation of eCS in other organs and systems. However, it is important to highlight that in all clinical studies cited, patients with a history of psychotic disorder were excluded from the study.

## Discussion

Chronic neuropathic pain is a pathology that affects not only many individuals worldwide, but also their caregivers (Ojeda et al., 2014). Even though opioids are the main pharmacotherapeutic approach to reduce symptoms associated with chronic neuropathic pain, several reports describe its potential of inducing addiction and, as consequence, increasing mortality (Hser et al., 2015). In order to avoid side effects similar to those induced by opioids and have increased success in managing pain, new molecular targets are being continuously investigated for treatment of neuropathic chronic pain.

In this review, we describe how the eCS modulates the pathophysiological processing of pain, focusing mostly on animal models of chronic neuropathic pain and clinical trials. Several strategies have been developed to assess eCS role in chronic neuropathic pain management, such as the use of synthetic cannabinoid receptors agonists and degradation enzyme inhibitors, which have shown promising results in animal models. Unfortunately, it is not always possible to translate pre-clinical data into successful clinical application. As an example, the use of FAAH inhibitors, which showed positive results in many animal models, led to severe adverse effects and even death of a volunteer in a clinical trial (Mallet et al., 2016).

The use of Cannabis-based products is recommended as extracts, for instance Sativex^®^. Although some synthetic cannabinoids may also be indicated for pain treatment, such as Dronabinol and Nabilone, the use of medicinal *Cannabis* sp. has recently increased substantially, improving pain management and inducing fewer side effects (Mücke et al., 2018). Despite this evidence, the IASP states that it lacks reliable clinical studies about the use of phytocannabinoids and medicinal *Cannabis* sp. to treat chronic pain (IASP Presidential Task Force on Cannabis and Cannabinoid Analgesia, 2021). Besides, there is little pre-clinical research that investigates the effect of *Cannabis* sp. extracts, the latter more commonly used by patients.

The role of eCS as a pharmacological target and the advantages of using medicinal *Cannabis sp.* to treat pain is remarkable, as described in this review. However, further investigation must be performed using animal models and in clinical practice to understand more efficient ways to modulate the eCS in humans and to identify how the entourage effect may contribute to the potential of Cannabis-based treatments in pain management.

## Author Contributions

RC, AA, YP-C, and PT wrote different sections of the review. BF also designed the original figures from this manuscript. RM and LS also provided the intellectual assistance, reviewed, and corrected the manuscript. All authors performed the literature revision need for this review and approved the submitted version.

## Conflict of Interest

The authors declare that the research was conducted in the absence of any commercial or financial relationships that could be construed as a potential conflict of interest.

## Publisher’s Note

All claims expressed in this article are solely those of the authors and do not necessarily represent those of their affiliated organizations, or those of the publisher, the editors and the reviewers. Any product that may be evaluated in this article, or claim that may be made by its manufacturer, is not guaranteed or endorsed by the publisher.

## Abbreviations

2-AG: 2-arachidonoylglycerol
AEA: anandamide
ASR-9: aggregated 9-factor symptom relief
ATP: adenosine triphosphate
cAMP: cyclic adenosine monophosphate
CB1R: cannabinoid receptor type 1
CB2R: cannabinoid receptor type 2
CBC: cannabichromene
CBD: cannabidiol
CBDV: cannabidivarin
CBN: cannabinol
CNS: central nervous system
COX-2: cyclooxygenase-2
CYP: cytochrome p450
DRG: dorsal root ganglia
EAAT2: excitatory amino acid transporter 2
eCBs: endocannabinoids
eCS: endocannabinoid system
eEPSC: evoked excitatory postsynaptic currents
FAAH: fatty acid amino hydrolase
GABA: γ -aminobutyric acid
IASP: International Association for the Study of Pain
IL-17: interleukin 17
IL-1 β: interleukin 1 β
IL-6: interleukin 6
MAGL: monoacylglycerol lipase
NF- κ b: nuclear factor κ B
P2X4 - P2X: purinoceptor 4
p38 MAPK: p38 mitogen-activated protein kinase
PDQ7: Pain Detect Questionnaire 7
PEA: palmitoylethanolamide
PNS: peripheral nervous system
THCV: Δ ^9^-tetrahydrocannabivarin
TNF- α: tumor necrosis factor- α
TRP: transient receptor potential
TRPA1: transient receptor potential subfamily A member 1
TRPM8: transient receptor potential subfamily M member 8
TRPV1: transient receptor potential subfamily V member 1
Δ ^9^-THC: Δ ^9^-tetrahydrocannabinol.
